# Iron Withdrawal with DIBI, a Novel 3-Hydroxypyridin-4-One Chelator Iron-Binding Polymer, Attenuates Macrophage Inflammatory Responses

**DOI:** 10.34172/apb.2023.040

**Published:** 2022-01-02

**Authors:** Javad Ghassemi-Rad, Wasundara Fernando, Bruce E. Holbein, David W. Hoskin

**Affiliations:** ^1^Department of Pathology, Faculty of Medicine, Dalhousie University, Halifax, Nova Scotia B3H 4R2, Canada.; ^2^Department of Microbiology and Immunology, Faculty of Medicine, Dalhousie University, Halifax, Nova Scotia B3H 4R2, Canada.; ^3^Department of Surgery, Faculty of Medicine, Dalhousie University, Halifax, Nova Scotia B3H 4R2, Canada.

**Keywords:** Cytokines, Inflammation, Iron, Macrophages, Reactive oxygen species, Signal transduction

## Abstract

***Purpose:*** Iron is an essential trace element for the inflammatory response to infection. In this study, we determined the effect of the recently developed iron-binding polymer DIBI on the synthesis of inflammatory mediators by RAW 264.7 macrophages and bone marrow-derived macrophages (BMDMs) in response to lipopolysaccharide (LPS) stimulation.

***Methods:*** Flow cytometry was used to determine the intracellular labile iron pool, reactive oxygen species production, and cell viability. Cytokine production was measured by quantitative reverse transcription polymerase chain reaction and enzyme-linked immunosorbent assay. Nitric oxide synthesis was determined by the Griess assay. Western blotting was used to assess signal transducer and activator of transcription (STAT) phosphorylation.

***Results:*** Macrophages cultured in the presence of DIBI exhibited a rapid and significant reduction in their intracellular labile iron pool. DIBI-treated macrophages showed reduced expression of proinflammatory cytokines interferon-β, interleukin (IL)-1β, and IL-6 in response to LPS. In contrast, exposure to DIBI did not affect LPS-induced expression of tumor necrosis factor-α (TNF-α). The inhibitory effect of DIBI on IL-6 synthesis by LPS-stimulated macrophages was lost when exogenous iron in the form of ferric citrate was added to culture, confirming the selectivity of DIBI for iron. DIBI-treated macrophages showed reduced production of reactive oxygen species and nitric oxide following LPS stimulation. DIBI-treated macrophages also showed a reduction in cytokine-induced activation of STAT 1 and 3, which potentiate LPS-induced inflammatory responses.

***Conclusion:*** DIBI-mediated iron withdrawal may be able to blunt the excessive inflammatory response by macrophages in conditions such as systemic inflammatory syndrome.

## Introduction

 Infection triggers a protective inflammatory response that is usually tightly regulated to prevent excessive damage to organs and tissues during pathogen elimination.^1^ Macrophages are key players in the inflammatory process by virtue of their ability to phagocytose and kill microorganisms, present antigen, produce pro-inflammatory and immunomodulatory cytokines, and regulate plasma iron.^2,3^ Iron is an indispensable co-factor in many important biological processes such as erythropoiesis, DNA synthesis and repair, and mitochondrial function, all of which involve iron-containing and iron-sequestering proteins.^4^ Iron also plays essential roles in innate immune cell function and the response to infection. Lipopolysaccharide (LPS)-stimulated macrophages produce hepcidin, which decreases iron absorption and suppresses the release of iron from macrophages, to withhold iron from invading bacteria and thereby limit their iron-dependent growth.^5,6^ Iron availability also impacts macrophage expression of proinflammatory cytokines and phosphorylation of Janus kinase (JAK)/signal transducer and activator of transcription (STAT) proteins involved in cytokine receptor signaling and the potentiation of inflammatory responses,^7-9^ as well as the synthesis of toxic reactive oxygen species (ROS) and reactive nitrogen species involved in macrophage killing of phagocytosed microorganisms.^10^ However, the dysregulation of iron homeostasis during an inflammatory response can result in oxidative stress and contribute to the pathogenesis of systemic inflammatory syndrome and several lung diseases.^11,12^

 Iron chelation has been proposed as a treatment for inflammatory disorders such as systemic inflammation associated with sepsis and lung inflammation in allergic rhinitis.^13-16^ However, the iron chelators that are currently approved for clinical use have several shortcomings.^17^ DIBI is a novel cell-impermeable and water-soluble iron-chelating compound created by co-polymerization of an active pyridinone chelating monomer 3-hydroxy-1-(β-methacrylamido-ethyl)-2-methyl-4(1H)-pyridinone with the structural monomer vinyl pyrrolidone to form a co-polymer of approximately 12 kDa molecular weight.^18^ DIBI was designed to have high selectivity and strong binding to Fe^3+^ iron.^19^ Moreover, DIBI shows no adverse effects, in terms of weight change and various hematological measurements and tissue histological examinations, in rats following oral administration of DIBI at up to 1000 mg/kg/day over 14 days.^19^ In this study, we determined the capacity of DIBI to blunt in vitro LPS-driven synthesis of inflammatory mediators by mouse bone marrow-derived macrophages (BMDMs) and the RAW 264.7 macrophage cell line to assess DIBI’s potential for use as an anti-inflammatory drug. Macrophages are involved in the inflammatory response through cytokine production/release and play a key role in regulating iron metabolism. It is therefore important to investigate the interrelationship of iron and the inflammatory response to LPS in macrophages.^20^

## Materials and Methods

###  Chemicals and reagents

 DIBI and polyvinyl pyrrolidone (PVP) were provided by Chelation Partners Inc. (Halifax, NS). Luminata^TM^ Forte Western horseradish peroxidase (HRP) (cat. # WBLUF0100), Griess reagent (cat. # G4410), β-mercaptoethanol (cat. # M6250), phosphate buffered saline (PBS) (cat. # P5493), phenylmethylsulfonyl fluoride (PMSF) (cat. # P7626), sodium deoxycholate (cat. # D6750), aprotinin (cat. # A1153), leupeptin (cat. # L2884), sodium fluoride (NaF) (cat. # S7920), sodium nitrate (cat. # S5506), pepstatin A (cat. # P5318), dithiothreitol (DTT) (cat. # D0632), 7-aminoactinomycin D (7-AAD) (cat. # A9400), and LPS (cat. # L2630) were purchased from Sigma Aldrich (Oakville, ON). Recombinant murine IL-6 (cat. # 216-16) and IFN-⍺ (cat. # AF-315-05) were purchased from PeproTech (Dollard des Ormeaux, QC). Fetal bovine serum (FBS) (cat. # 12483020), 5-(and-6)-chloromethyl-2’,7’-dichlorodihydrofluorescein diacetate, acetyl ester (CM-H_2_DCFDA) (cat. # C6827), calcein acetoxymethyl ester (calcein-AM) (cat. # C3099), RPMI 1640 medium with (cat. # 11875119) and without phenol-red (cat. # 32404014), TrypLE^TM^ Express (cat. # 11875093), L-glutamine (cat. # 25030), penicillin/streptomycin (cat. # 15140), and N-2-hydroxyethylpiperazine-N-2-ethane sulfonic acid (HEPES) (cat. # 15630) were purchased from Thermo Fisher Scientific (Burlington, ON). Sodium chloride (NaCl) (cat. # SOD002), acrylamide/bis-acrylamide (29:1, 30%) (cat. # ACR009), ammonium persulfate (APS) (cat. # AMP001), sodium dodecyl sulfate (SDS) (cat. # SDS002), Tween-20 (cat. # TWN508), glycine (cat. # GLN001), tetramethylethylenediamine (TEMED) (cat. # TEM001), ethylene glycol tetraacetic acid (EGTA) (cat. # EGT202), and ethylene diamine tetraacetic acid (EDTA) (cat. # EDT222) were purchased from BioShop Canada Inc. (Burlington, ON).

###  Antibodies

 Anti-phospho-STAT1 (Tyr 701) rabbit antibody (Ab) (cat. # 9167), anti-phospho-STAT3 (Tyr 705) rabbit Ab (cat. # 9131), anti-STAT1 rabbit Ab (cat. # 9175), anti-STAT3 mouse Ab (cat. # 9139), anti-rabbit IgG HRP-linked Ab (cat. # 7074), anti-mouse IgG HRP-linked Ab (cat. # 7076), and anti-β-actin HRP-linked Ab (cat. # 5125) were all purchased from Cell Signaling Technology Inc. (Beverly, MA). All primary Abs were reactive against mouse proteins.

###  Cell line and culture conditions

 The RAW 264.7 mouse macrophage cell line was purchased from ATCC (Manassas, VA) and maintained at 37℃ in a humidified 5% CO_2_ incubator using RPMI 1640 medium supplemented with 5% heat inactivated FBS, 2mM L-glutamine, 100 µg/mL streptomycin, 100 U/mL penicillin, and 5 mM HEPES buffer (pH 7.4), referred to as complete medium. Cells were passaged using a 25 cm cell scraper (VWR, Mississauga, ON).

###  Isolation of murine BMDMs

 Female C57BL/6 mice were purchased from Charles River Laboratories (LaSalle, QC) and housed in the Carlton Animal Care Facility at Dalhousie University (Halifax, NS). Mice were fed a standard diet of rodent chow and water supplied ad libitum. All procedures using mice were approved by the Dalhousie University Committee on Laboratory Animals (animal protocol # 18-056a), in accordance with guidelines provided by the Canadian Council on Animal Care. Mice were sacrificed by cervical dislocation at 8-10 weeks of age and bone marrow was harvested from tibias and femurs via aseptic technique. Bone marrow cells were homogenized in cold PBS (pH 7.2) and red blood cells were removed by hypo-osmotic shock. Bone marrow cells were then cultured at 37°C in a humidified 5% CO_2_ incubator using complete medium supplemented with 15% L929-conditioned medium containing macrophage colony-stimulating factor for 7 days to obtain mature BMDMs.^21^ Additional culture medium was added to BMDMs on days 3 and 6. BMDMs were harvested on day 7 for use in experiments.

###  Cell viability measurement by 7-AAD staining

 Macrophages were cultured for 24 hours in the absence or presence of 200 µM DIBI and then collected and stained with 10 µg/mL 7-AAD in PBS for 5 minutes. Cells were then washed with PBS and analyzed (1 × 10^4^ cell counts/sample) using the FL1 channel of a BD FACSCanto^TM^ flow cytometer (BD Biosciences, Mississauga, ON). Data were processed using FCS Express software (version 3.0, De Novo Software, Thornhill, ON).

###  Measurement of the intracellular labile iron pool

 RAW 264.7 cells were seeded into a 6-well plate at 2.5 × 10^5^ cells/well and cultured overnight to allow cells to adhere. The following day, cells were treated with 0.5 µM calcein-AM and cultured for 30 minutes at 37°C. The fluorescence of calcein-AM is quenched by intracellular iron.^22^ Cells were then washed with room temperature PBS and treated with complete medium alone or complete medium containing 200 µM DIBI or 1.28 mg/mL PVP. Cells that were not exposed to calcein-AM served as an additional control. After 4, 24, and 48 hours of culture cells were harvested and analyzed (1 × 10^4^ cell counts/sample) using the FL1 channel of a BD FACSCanto^TM^ flow cytometer (BD Biosciences, Mississauga, ON). Data were processed using FCS Express software (version 3.0, De Novo Software, Thornhill, ON).

###  Enzyme linked immunosorbent assay (ELISA)

 RAW 264.7 cells or BMDMs in complete medium were seeded into 24-well plates at 1 × 10^5^ cells/well and cultured overnight. The following day cells were treated with complete medium alone or complete medium containing the indicated concentrations of DIBI or PVP in the presence or absence of 100 ng/mL LPS. In some experiments, RAW 264.7 cells received 100 ng/mL LPS and 1000 µM of ferric citrate (Fe) in the presence or absence of 100 µM DIBI. After 24 hours of culture cell-free supernatants were harvested for detection of tumor necrosis factor-α (TNF-α) and IL-6 using sandwich ELISA Ready-SET-Go!^Ⓡ^ kits (TNF-⍺, cat. # BMS607HS; IL-6, cat. # KMC0061) from Thermo Fisher Scientific (Burlington, ON), according to manufacturer’s protocol. The colorimetric reaction was stopped with 50 µL of 0.3 M sulfuric acid and the absorbance at 450 nm was determined with an Expert 96 microplate reader (Biochrome ASYS, Cambridge, UK).

###  Quantitative reverse transcription polymerase chain reaction ( qRT -PCR)

 RAW 264.7 cells or BMDMs in complete medium were seeded into 6-well plates at 2.5 × 10^5^ cells/well (RAW 264.7 cells) or 5 × 10^5^ cells/well (BMDMs) and cultured overnight. The following day, cells were treated with complete medium alone or complete medium containing the indicated concentrations of DIBI or PVP in the absence or presence of 100 ng/mL LPS. After 6 hours treatment, RNA was isolated using a RNeasy Mini kit (cat. # 74104, Qiagen, Valencia, CA), according to manufacturer’s instructions. Quantity and quality of RNA were determined with a NanoVue Plus Spectrometer (GE Healthcare Life Sciences, Piscataway Township, NJ). Approximately 100 ng RNA from BMDMs or 500 ng RNA from RAW 264.7 cells were reverse transcribed to cDNA in a final volume of 10 µL using an iScript^TM^ cDNA synthesis kit (cat. # 1728890, Bio-Rad, Hercules, CA), according to manufacturer’s instructions. The reaction was incubated in a Bio-Rad T100^TM^ Thermocycler for 5 minutes at 25°C, 30 minutes at 42°C, and 5 minutes at 85°C. The cDNA samples were kept at -20°C prior to use. Reaction mixtures for qRT-PCR contained sample cDNA, primer mix (10 µM of forward and reverse primer), nuclease-free water, and SsoFast EvaGreen^TM^ SupermixⓇ (cat. # 1725201, Bio-Rad, Hercules, CA) in a 1:1:3:5 ratio, respectively. A 10 µL volume of each mixture was added to triplicate wells of a Multiplate^TM^ 96-well unskirted polypropylene PCR plate (Bio-Rad, Hercules, CA) with a Microseal ‘B’ Adhesive sealing film (Bio-Rad, Hercules, CA). The plate was centrifuged at 500 rcf for 10 seconds and placed in CFX Connect^TM^ Real-Time PCR detection system (Bio-Rad, Hercules, CA) using the following reaction steps: 30 minutes at 95°C, 40 cycles of 5 seconds at 95°C, and 30 seconds at primer-specific annealing temperatures. Forward and reverse primer sets, and annealing temperatures (see Table 1) were obtained from Integrated DNA Technologies, Inc. (Coralville, IA). Primers were confirmed on Primer-BLAST database and tested for optimal activity and efficacy according to MIQE guidelines.^23^ Murine GAPDH was used as a reference gene.

**Table 1:** Primer set sequences

**Primer set**	**Forward primer**	**Reverse primer**	**Annealing temperature**
IFN-β	5’-GCC ATC CAA GAG ATG CTC CA-3’	5’-GGT ACC TTT GCA CCC TCC AG-3’	61.4℃
IL-1β	5’-AGC TTC AGG CAG GCA GTA TC-3’	5’-AAG GTC CAC GGG AAA GAC AC-3’	55.7℃
IL-6	5’-TCC AGT TGC CTT CTT GGG AC-3’	5’-AGT CTC CTC TCC GGA CTT GT-3’	55℃
GAPDH	5’-CCA CTT CAA CAG CAA CTC CCA CTC TTC-3	5’-TGG GTG GTC CAG GGT TTC TTA CTC CTT-3’	_*_
TNF-⍺	5’-CAT CTT CTC AAA ATT CGA GTG ACA A-3’	5’-TGG GAG TAG ACA AGG TAC AAC CC-3’	56℃

* The annealing temperature for the GAPDH primer set corresponds to the annealing temperature of the primer set for the cytokine gene of interest. The primer set for GAPDH was tested at different annealing temperatures to confirm efficient amplification and production of a single product at each temperature.

###  Reactive oxygen species (ROS) measurement 

 The CM-H_2_DCFDA assay was used to measure intracellular ROS production.^24^ RAW 264.7 cells were seeded into 6-well plates at 2.5 × 10^5^ cells/well and cultured overnight. The following day, cells were cultured with complete medium alone or complete medium containing 200 μM DIBI or 1.28 mg/mL PVP in the absence or presence of 1 µg/mL LPS. After 24 hours culture, the cells were washed with PBS and resuspended in FBS- and phenol red-free medium containing 10 µM of CM-H_2_DCFDA and incubated in dark at 37°C for 30 minutes. Cells were then harvested with TrypLE^TM^ Express and washed with room temperature PBS. Cells (1 × 10^4^ cell counts/sample) were analyzed via the FL1 channel of a BD FACSCalibur^TM^ flow cytometer (BD Biosciences, Mississauga, ON). Data were processed using FCS Express software (version 3.0, De Novo Software, Thornhill, ON).

###  Nitric oxide (NO) measurement 

 The Griess reaction was used for colorimetric determination of NO production.^25^ RAW 264.7 cells were seeded into 24-well plates at 1 × 10^5^ cells/well and cultured overnight. The following day, cells were washed with room temperature PBS and resuspended in complete medium alone (phenol red-free) or complete medium alone (phenol red-free) containing 200 µM DIBI or 1.28 mg/mL PVP in the absence or presence of 100 ng/mL LPS. After 24 hours of culture, cell-free supernatants were harvested and transferred to quadruplicate wells of a flat bottom 96-well plate and mixed with Griess reagent in a 1:1 ratio. A standard curve was generated using different concentrations of sodium nitrate in phenol red-free complete medium. Plates were incubated at room temperature in the dark for 15 minutes prior to measuring absorbance at 570 nm with an Expert 96 microplate reader (Biochrome ASYS, Cambridge, UK).

###  Western blot analysis

 RAW 264.7 cells were seeded into T-75 mm^2^ cell culture flasks at 1 × 10^6^ cells/flask and cultured for 36-48 hours until 80% confluency was achieved. Cells were then treated for 4 hours with 25 ng/mL of murine IL-6 or murine IFN-⍺ in the absence or presence or absence of 200 µM DIBI. Cells were pelleted and cell lysates were prepared by resuspending cells in RIPA buffer (1% Nonidet P-40, 0.25% sodium deoxycholate, 50 mM Tris-HCl, 150 mM NaCl, 5 mM of EDTA, 5 mM of EGTA at pH 7.5, with 5 µg/mL leupeptin, 5 µg/mL pepstatin A, 10 µg/mL aprotinin, 100 µM sodium orthovanadate, 1 mM DTT, 10 mM NaF, 10 µM PAO, and 1 mM PMSF) and incubating on ice for 15 minutes. Cellular debris were then removed by centrifugation (14 000 rcf at 4°C for 10 minutes). The protein concentration in cell lysates was determined using Bio-Rad protein assay kit (Hercules, CA). Equal amounts of protein were diluted with RIPA buffer and 3X SDS-polyacrylamide gel electrophoresis buffer (6% SDS, 30% glycerol, 15% β-mercaptoethanol, 0.01% bromophenol blue, and 200 mM Tris-HCl (pH 6.8)) in a 2:1 ratio, respectively. Proteins were denatured by heating at 95°C for 5 minutes and then stored at -80°C prior to use. Protein samples (15 µg) were run on a 10% SDS-polyacrylamide gel (10% acrylamide, 0.1% SDS, 125 mM Tris-HCl (pH 8.8), 0.15% TEMED, and 0.1% APS) with a 4% stacking gel (4% acrylamide, 0.1% SDS, 125 mM Tris-HCl (pH 8.8), 0.15% TEMED, and 0.1% APS) at 200 volts for 1 hour. SDS running buffer (0.1% SDS, 200 mg glycine, and 200 mM Tris-HCl (pH 8.3)) was used for electrophoresis at 200 volts for 1 hour. Proteins were then transferred to a nitrocellulose membrane using an iBlot^TM^ dry transfer system (Thermo Fisher Scientific, Burlington, ON) for 6.5 minutes. Membranes were soaked with 5% FBS in Tween-20 Tris-buffered saline (TTBS) (20 mM Tris-HCl (pH 6.8), 200 mM NaCl, and 0.05% Tween-20) for 1 hour at room temperature on a rocker. Primary Abs were used to probe for specific proteins at 4°C overnight. The following day, membranes were washed with TTBS for 30 minutes; TTBS was changed every 5 minutes. Then, membranes were incubated with HRP-conjugated secondary Ab for 2 hours at room temperature on a rocker. After washing for 30 minutes with TTBS, membranes were developed using a ChemiDoc Imaging system (Bio-Rad, Hercules, CA). Protein band intensities were quantified using Image Lab software (version 5.2, Bio-Rad, Hercules, CA).

###  Statistical analysis

 Data was analyzed with GraphPad Prism (Version 5.0, GraphPad Software, Inc., La Jolla, CA). One-way analysis of variance (ANOVA) with Tukey-Kramer or Bonferroni multiple comparisons post-tests, when appropriate, were used to determine statistical significance. At least three independent biological replicates were conducted for each experiment. Results were considered significant at *P* < 0.5.

## Results and Discussion

###  DIBI reduces the intracellular pool of labile iron in macrophages

**Figure 1:**
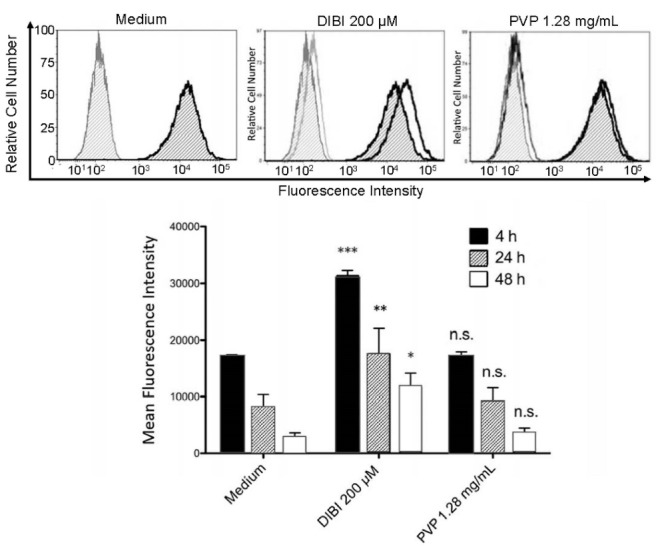
DIBI reduces the intracellular labile iron pool of macrophages. RAW 264.7 macrophages were loaded with 0.5 µM calcein-AM and plated into 6-well plates at 250.000 cells/well in medium alone or medium containing the indicated concentrations of DIBI or PVP. After 4, 24 and 48 hours of culture, cells were harvested, and fluorescence intensity was measured by flow cytometry. The histogram depicts data from a representative experiment. Mean fluorescence intensity values ± standard error of the mean (SEM) were determined from 3 independent experiments; * denotes statistical significance using two-way ANOVA and the Bonferroni post-test.

 The impact of DIBI on the availability of iron to macrophages was determined by comparing the intracellular pool of labile iron in RAW 264.7 macrophages that were cultured in the absence or presence of 200 μM DIBI. Staining with 7-AAD cell viability dye showed that 200 μM DIBI was not cytotoxic for RAW 264.7 macrophages (control cells: 95 ± 3% viable after 24 hours culture; DIBI-treated cells: 87 ± 5% viable after 24 hours culture). As shown in Figure 1, RAW 264.7 macrophages that were cultured in the presence of DIBI showed a significant decrease in the intracellular pool of labile iron because of iron chelation in the culture medium, as determined by flow cytometric analysis of calcein-AM-labeled cells. This effect was specific to the iron-chelating activity of DIBI since an equivalent concentration of similar molecular weight PVP molecules that are the structural backbone component of the DIBI co-polymer did not reduce the intracellular pool of labile iron in RAW 264.7 macrophages. The DIBI co-polymer has been previously characterized.^26^ A similar decrease in the labile iron pool occurs when MDA-MB-468 breast cancer cells are cultured in the presence of DIBI.^27^ Cell-impermeable DIBI therefore effectively reduces the intracellular pool of labile iron in immune cells and non-immune cells, presumably by providing an extracellular high affinity sink for iron. In this regard, DIBI possesses an iron binding affinity approximately three orders of magnitude higher than other chelators such as deferiprone and, correspondingly, can effectively strip iron from deferiprone.^22^

###  DIBI-mediated iron withdrawal suppresses excess macrophage synthesis of proinflammatory IL-1β and IL-6

**Figure 2:**
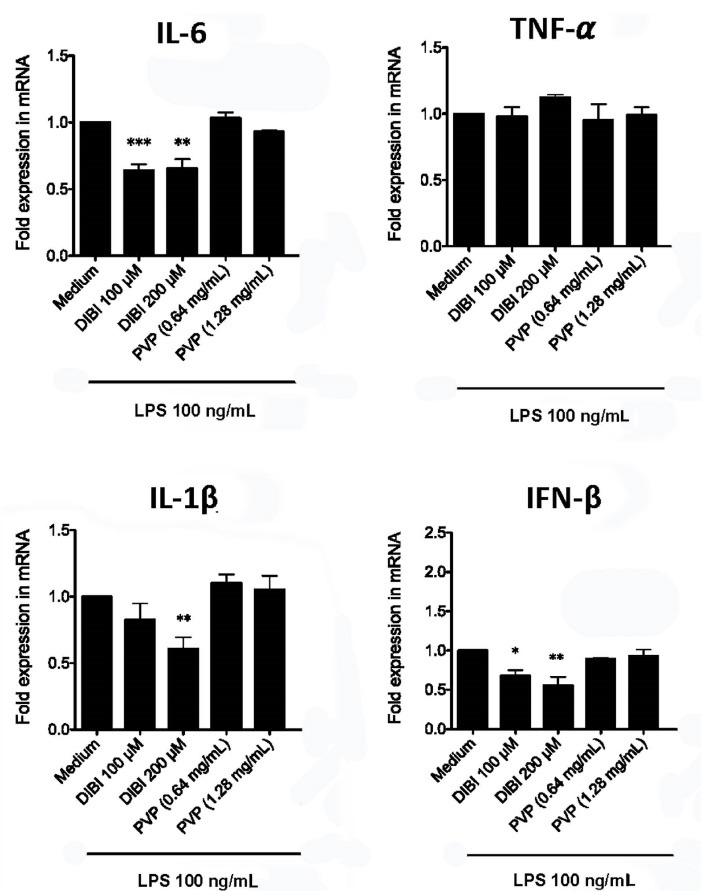
DIBI inhibits RAW 264.7 macrophage expression of IL-1β, IL-6 and IFN-β mRNA but not TNF-α mRNA. RAW 264.7 macrophages were cultured for 6 hours with medium alone or medium containing the indicated concentrations of DIBI, PVP, or DFP plus 100 ng/mL LPS. Cytokine mRNA expression relative to the medium-only control was determined by qRT-PCR. Little or no cytokine-encoding mRNAs were not detected in the absence of LPS stimulation (data not shown). Data shown are the mean fold expression ± SEM of 3 independent experiments; * denotes statistical significance using two-way ANOVA and the Bonferroni post-test.

**Figure 3:**
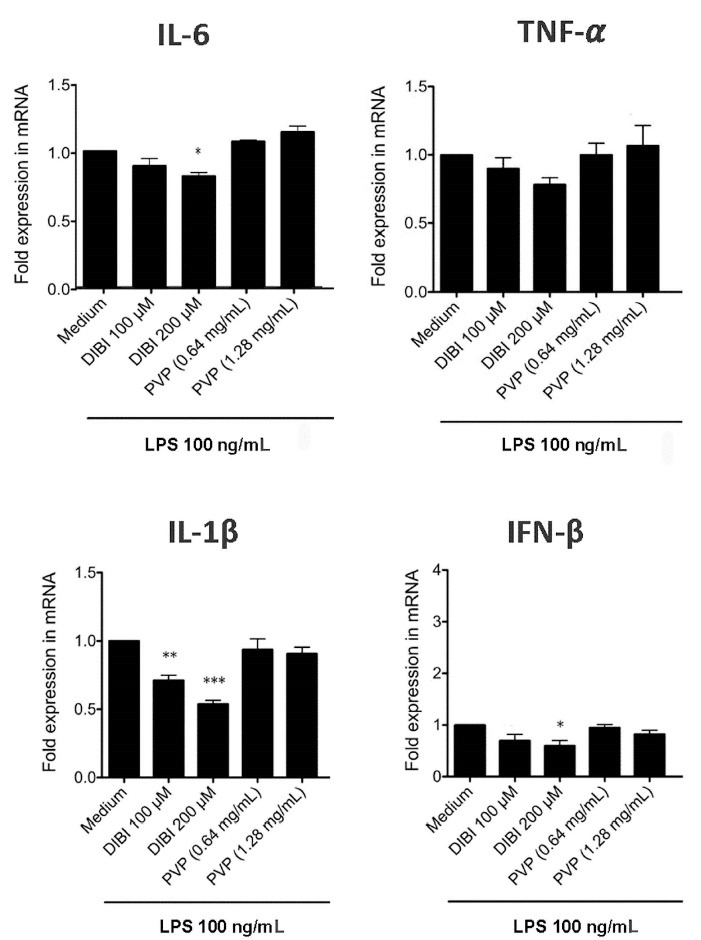
DIBI inhibits BMDM expression of IL-1β, IL-6 and IFN-β mRNA but not TNF-α mRNA. BMDMs were cultured for 6 hours with medium alone or medium containing the indicated concentrations of DIBI, PVP, or DFP plus 100 ng/mL LPS. Cytokine mRNA expression relative to the medium-only control was determined by qRT-PCR. Little or no cytokine-encoding mRNAs were not detected in the absence of LPS stimulation (data not shown). Data shown are the mean fold expression ± SEM of 3 independent experiments; * denotes statistical significance using two-way ANOVA and the Bonferroni post-test.

 RT-qPCR and ELISA were used to assess the effect of DIBI-mediated iron withdrawal on proinflammatory cytokine synthesis by LPS-stimulated macrophages. Figure 2 shows that RAW 264.7 macrophages that were stimulated with LPS in the presence of DIBI had reduced synthesis of mRNA coding for the inflammation-promoting cytokines IL-1β and IL-6, as well as IFN-β, in comparison to RAW 264.7 macrophages that were stimulated with LPS in the absence of DIBI. This inhibitory effect of DIBI on the expression of cytokine-encoding mRNA was not seen with PVP alone. In contrast, DIBI did not affect TNF-α mRNA synthesis by LPS-stimulated RAW 264.7 macrophages. Similar results were obtained using LPS-stimulated BMDMs (Figure 3), indicating that the inhibitory effect of DIBI on IFN-β, IL-1β and IL-6 mRNA expression, as well as the inability of DIBI to affect TNF-α mRNA synthesis, was not restricted to the RAW 264.7 macrophage line. In addition, normal TNF-α synthesis by DIBI-treated macrophages confirmed that DIBI did not adversely impact cell viability.

**Figure 4:**
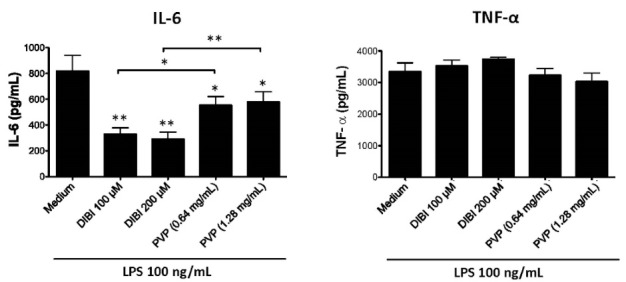
DIBI inhibits RAW 264.7 macrophage synthesis of IL-6 but not TNF-α. RAW 264.7 macrophages were cultured for 24 hours in medium alone or medium containing the indicated concentrations of DIBI or PVP in the absence or presence of 100 ng/mL LPS. At the end of culture, cell-free supernatants were collected, and ELISA was used to measure IL-6 and TNF-α. Data shown are the mean values ± SEM of 3 independent experiments. RAW 264.7 macrophages that were not stimulated with LPS showed little or no cytokine production that did not change with the addition DIBI or PVP (data not shown). Statistical significance was determined by ANOVA with the Tukey multiple comparisons post-test; * *P* < 0.05; ** *P* < 0.01.

**Figure 5:**
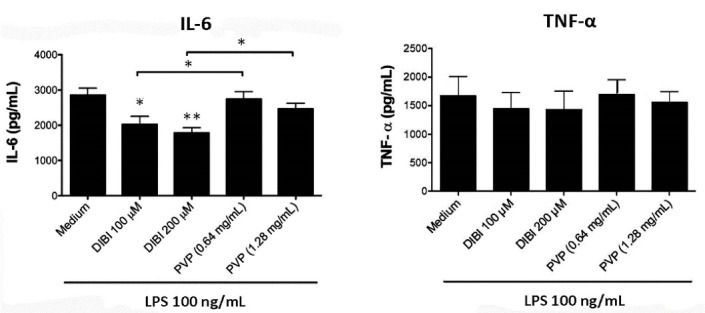
DIBI inhibits BMDM synthesis of IL-6 but not TNF-α. BMDMs were cultured for 24 hours in medium alone or medium containing the indicated concentrations of DIBI or PVP in the absence or presence of 100 ng/mL LPS. At the end of culture, cell-free supernatants were collected and ELISA was used to measure IL-6 and TNF-α. Data shown are the mean values ± SEM of 3 independent experiments. BMDMs that were not stimulated with LPS showed little or no cytokine production that did not change with the addition DIBI or PVP (data not shown). Statistical significance was determined by ANOVA with the Tukey multiple comparisons post-test; * *P* < 0.05; ** *P* < 0.01.

 Consistent with the mRNA data, there was less IL-6 in cultures of DIBI-treated RAW 264.7 macrophages (Figure 4) and BMDMs (Figure 5), relative to control cultures, following stimulation with LPS. Interestingly, PVP alone had a slight inhibitory effect on IL-6 production by LPS-stimulated RAW264.7 macrophages; however, this effect was not seen with LPS-stimulated BMDMs. LPS-induced synthesis of TNF-α by RAW 264.7 macrophages and BMDMs was not affected by DIBI treatment. Interestingly, the iron-chelator deferiprone suppresses TNF-α synthesis, as well as IL-1β synthesis by LPS-stimulated human macrophages.^28^ It is possible that, unlike membrane-impermeable DIBI, membrane-permeable deferiprone inhibits TNF-α synthesis by macrophages due to its ability to cause iron imbalances between mitochondria and the cytosol, resulting in an anti-inflammatory immunometabolic switch. Our finding that DIBI did not affect TNF-α expression by macrophages is of importance because macrophages should not be completely immunosuppressed but rather remain capable of mounting a protective response. DIBI did inhibit excess IL-1β, IL-6, and IFN-β expression by LPS-stimulated macrophages. This has significance as these cytokines are important drivers of inflammation, but excess production has the potential to cause tissue damage and disease.^29-31^ Administration of DIBI also reduces plasma IL-1β and IL-6 in a mouse model of sepsis.^16^ Our findings regarding the importance of available iron in the generation of an inflammatory response are in line with a report that iron-loaded macrophages show enhanced LPS-induced inflammatory responses.^32^

**Figure 6:**
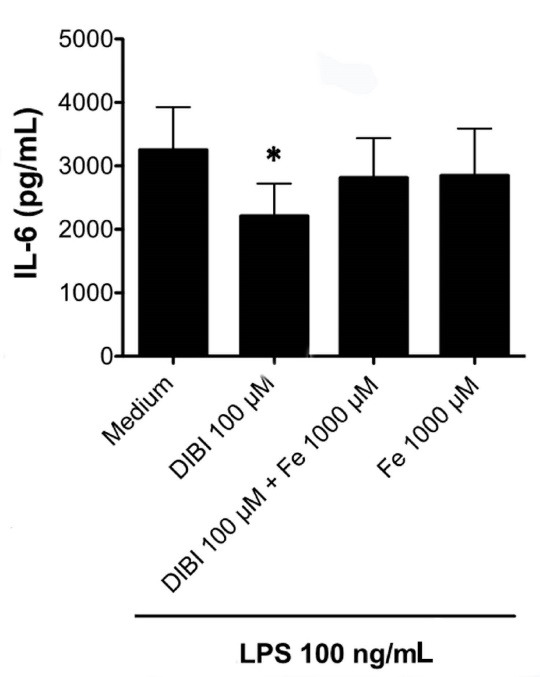
Exogenous iron ablates the inhibitory effect of DIBI on macrophage synthesis of IL-6. RAW 264.7 macrophages were cultured for 24 hours with medium alone or medium containing 200 μM DIBI and/or iron (Fe) as 1000 μM ferric citrate in the absence or presence of 100 ng/mL LPS. Cell-free supernatants were collected, and IL-6 was measured by ELISA. Data shown are the mean ± SEM of 4 independent experiments. RAW 264.7 macrophages that were not stimulated with LPS showed little or no cytokine production that did not change with the addition DIBI or iron (data not shown). Statistical significance was determined by ANOVA with the Tukey multiple comparisons post-test; * *P* < 0.05.

Figure 6 shows that the inhibitory effect of DIBI on IL-6 synthesis by LPS-stimulated RAW 264.7 macrophages was eliminated by the addition of exogenous iron in the form of iron (III) citrate. This finding, together with the DIBI-induced reduction in the labile iron pool of macrophages (Figure 1), confirms that DIBI-mediated inhibition of IL-6 synthesis and, presumably, the synthesis of IFN-β and IL-1β were specifically the result of iron withdrawal.

###  DIBI-mediated iron withdrawal suppresses ROS and NO production by macrophages

**Figure 7:**
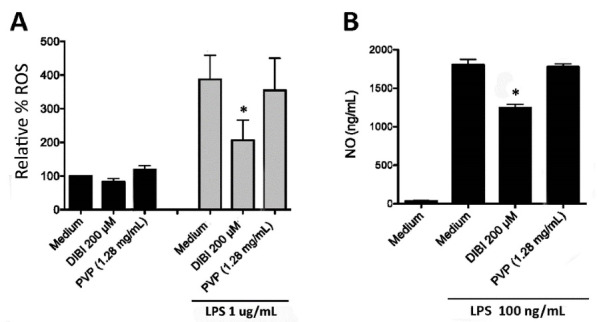
DIBI reduces macrophage synthesis of ROS and NO. (A) RAW 264.7 macrophages were stained with 10 µM CM-H_2_DCFDA and cultured for 24 hours with medium alone or medium containing 200 μM DIBI or 1.28 mg/mL PVP in the absence or presence of 1 μg/mL LPS. Cell fluorescence was then measured by flow cytometry. Data are shown as the mean percentage of ROS formation ± SEM of 3 independent experiments. (B) RAW 264.7 macrophages were cultured for 24 hours with medium alone or medium containing 200 μM DIBI or 1.28 mg/mL PVP in the absence or presence of 100 ng/mL LPS. At the end of culture, NO in cell-free culture supernatants was measured by colorimetric Griess assay and calibrated using a standard curve. Data shown are the mean NO ± SEM of 3 independent experiments. (A, B) Statistical significance was determined by two-way ANOVA and the Bonferroni post-test; * *P* < 0.05.

 To further characterize the impact of DIBI-mediated iron withdrawal on the inflammatory response of macrophages, we assessed LPS-stimulated macrophage production of ROS and NO, both of which are mediators of inflammation. Flow cytometric analysis of CM-H_2_DCFDA-stained macrophages was used to measure intracellular ROS accumulation. As shown in Figure 7A, LPS-stimulated RAW 264.7 macrophages showed a 4-fold increase in ROS compared to unstimulated control cells. In this experiment a higher concentration (1 μg/mL) of LPS was used to trigger vigorous synthesis of ROS. Treatment with 200 μM DIBI, but not a similar concentration of PVP, reduced ROS production by LPS-stimulated RAW 264.7 macrophages. ROS that are important for host defence and contribute to inflammatory disease are produced via the electron transport chain of mitochondria, cytochrome P450, and NADPH oxidases.^33^ Since the iron-dependent Fenton reaction is important for ROS generation, including the highly toxic hydroxyl radical, it follows that intracellular ROS was reduced in LPS-stimulated macrophages that were treated with DIBI. Importantly, DIBI did not completely inhibit ROS production; rather, DIBI dampened excess ROS production. This could have significance given macrophage ROS-associated killing of phagocytosed bacterial pathogens is also important in host defence. Decreased ROS production by LPS-stimulated macrophages in the presence of DIBI may contribute to the reduction in IL-1β, IL-6, and IFN-β synthesis since mitochondrial ROS promote LPS-induced cytokine synthesis.^34^ These various results are consistent with DIBI dampening but not fully blocking the excess LPS-induced inflammatory response, i.e., excess ROS and cytokine production.

 The colorimetric Griess assay was used to measure NO in cell culture supernatants. Figure 7B shows that LPS stimulation of RAW 264.7 macrophages resulted in a 43-fold increase in NO, which was reduced by a third in the presence of 200 μM DIBI. Treatment with an equivalent concentration of PVP failed to affect LPS-induced NO production by RAW 264.7 macrophages. NO combines with ROS such as superoxide to form reactive nitrogen species that contribute to the inflammatory response.^33^ Given this; reduced levels of ROS might be expected to result in reduced levels of NO. Although the full mechanism by which DIBI-mediated iron withdrawal suppresses NO synthesis is not yet clear, our findings suggest that the formation of reactive nitrogen species by LPS-stimulated macrophages should be diminished because of reduced ROS.

###  DIBI-mediated iron withdrawal inhibits cytokine-induced STAT1 and STAT3 phosphorylation

 We also determined the effect of DIBI-mediated iron withdrawal on signaling pathways involved in the macrophage inflammatory response. LPS-mediated triggering of Toll-like receptor 4 on macrophages leads to the activation and nuclear localization of the transcription factor NF-κB, which is responsible for initiating the transcription of genes coding for the proinflammatory cytokines TNF-α and IL-6.^35^ However, western blot analysis and confocal microscopy did not show any effect of DIBI on LPS-induced NF-κB activation in RAW 264.7 macrophages (data not shown), which is consistent with the inability of DIBI-mediated iron withdrawal to affect TNF-α production by LPS-stimulated macrophages (Figures 4 and 5). It is possible that sufficient intracellular iron may remain available for NF-κB activation in DIBI-treated macrophages.

**Figure 8:**
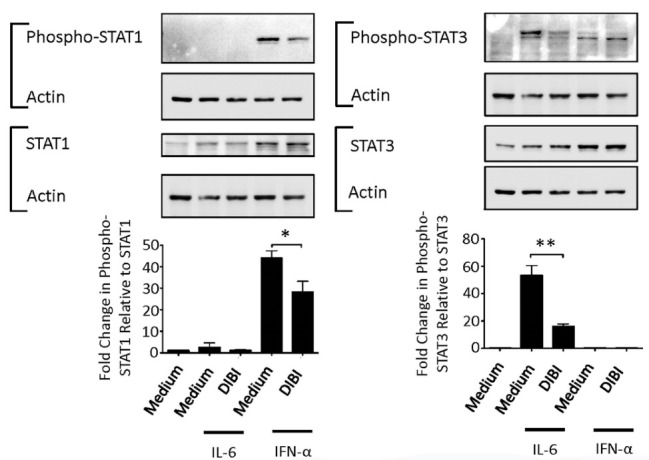
DIBI inhibits STAT1 and STAT3 signaling in macrophages. RAW 264.7 macrophages were cultured for 4 hours with medium alone or medium containing DIBI without or with IL-6 (0.5 ng/mL) or IFN-α (0.5 ng/mL). Cytosolic proteins were extracted for western blot analysis to measure phospho-STAT1/STAT3 and total STAT1/STAT3. Actin expression was determined to confirm equal protein loading. Representative blots are shown. Histograms show data from 3 independent experiments. Protein bands were normalized relative to actin prior to normalizing phospho-STAT1/STAT3 relative to total STAT1/STAT3 and presented as fold change in expression. Statistical significance was determined by ANOVA with the Tukey multiple comparisons post-test; * *P* < 0.05; ** for *P* < 0.001.

 STAT1 is needed for LPS-induced expression of certain proinflammatory proteins, with the notable exception of TNF-α,^36^ while STAT3 potentiates IL-6 synthesis via a cytokine amplification loop.^37^ We therefore determined the effect of DIBI-mediated iron withdrawal on STAT1 and STAT6 activation. Figure 8 shows that phosphorylation of cytokine receptor-associated STAT1 and STAT3 in response to IFN-α and IL-6, respectively, was decreased in the presence of 200 μM DIBI. Flow cytometric analysis revealed that concentrations of DIBI that inhibited STAT1 and STAT3 phosphorylation did not affect expression of type I IFN receptor and IL-6 receptor components by RAW 264.7 macrophages (data not shown). The inhibitory effect of DIBI on signal transduction associated with IFN-α/β and IL-6 receptors on RAW 264.7 macrophages accounts for reduced synthesis of IL-6 and other proinflammatory cytokines in the absence of any inhibition of NF-κB activation. Our findings are consistent with the reported inhibition of JAK/STAT signaling by the iron-chelators desferrioxamine and 2-2’-dipyridyl.^8^ Although the mechanism by which iron withdrawal inhibits STAT1 and STAT3 activation is not yet fully known, it is noteworthy that iron is involved in the inhibition of protein tyrosine phosphatases in macrophages.^38^ It follows that protein tyrosine phosphatase activity should increase under iron-limiting conditions, as occur in the presence of DIBI, which would in turn cause STAT1 and STAT3 dephosphorylation and subsequent reduced activation of these transcription factors.

## Conclusion

 DIBI-mediated iron withdrawal reduced the intracellular labile iron pool of macrophages and inhibited LPS-induced expression of IFN-β and inflammation-promoting IL-1β and IL-6, but not TNF-α. Down-regulation of STAT1 and STAT3 transcription factor activation may account for blunting the synthesis of IL-1β and IL-6. DIBI also reduced ROS and NO production by LPS-stimulated macrophages. We conclude that DIBI shows promise as an anti-inflammatory agent in situations where an excessive inflammatory response may cause disease.

## Acknowledgments

 This research was funded by the Natural Sciences and Engineering Research Council of Canada.

## Competing Interests

 BEH has a beneficial interest in Chelation Partners Inc. The other authors declare that they have no conflict of interests.

## Ethical Approval

 Not applicable.
